# Comparing the symmetry of upper eyelid following unilateral ptosis correction

**DOI:** 10.1186/s12886-021-02208-7

**Published:** 2021-12-20

**Authors:** Hasan Aytogan, Emre Ayıntap

**Affiliations:** Izmir Tepecik Training and Research Hospital, Yenisehir, Konak, Izmir, Turkey

**Keywords:** Ptosis, Eyelid symmetry, Ptosis correction, Levator procedures, Eyelid contour

## Abstract

**Background:**

Margin Reflex Distance 1(MRD 1) only describes the central height of upper eyelid and relies on the examiner’s experience and disregards eyelid contour abnormalities. Therefore MRD 1 may not be sufficient for an acceptable result to evaluate the outcomes of ptosis surgery. The primary purpose of this study was to assess outcomes of unilateral ptosis correction based on parameters including degree of symmetry, MRD 1, peak height of the upper lid, temporal and nasal ocular surface area, and temporal/nasal area ratio with an objective, quantitative, and repeatable method.

**Methods:**

This study was designed as a retrospective non-randomized case-control study. Medical records of the patients with unilateral ptosis between October 2015 and December 2020 were reviewed. Patients with unilateral ptosis who underwent surgical correction and levator function of 5 mm or greater were included in the study. Two groups were defined; ptotic eye was case group and contralateral eye was control group. Data analysis was performed Image J and Matlab softwares.

**Results:**

Thirty-four patients were included in the study. Mean age of patients was 58.8 ± 12.7 years (range 15–75 years). Mean follow-up time was 19.5 ± 7.3 months (range 8–40 months). Four patients were diagnosed with congenital ptosis and 30 patients aponeurotic ptosis. Mean preoperative degree of symmetry for overall eyelid contour was 36.6 ± 27.5% (range 1–92%). Mean postoperative degree of symmetry for overall eyelid contour was 72.4 ± 16.5% (range 55–92%). Temporal/Nasal (T/N) area ratios for contralateral normal eye was 1.19 pre-postoperative, and it was 1.11 preoperatively, 1.15 postoperatively for operated ptotic eye.

**Conclusions:**

This study primarily demonstrated a quantitative, objective, and repeatable method to investigate the degree of symmetry after eyelid surgeries. Secondly, this study suggested that T/N ratio may not be a reliable parameter to evaluate the eyelid symmetry.

**Supplementary Information:**

The online version contains supplementary material available at 10.1186/s12886-021-02208-7.

## Background

The primary outcome of ptosis surgery is traditionally an increased Margin Reflex Distance 1 (MRD1), which only describes the central height of the upper lid and relies on the examiner’s experience [1, 2]. As MRD1 disregards eyelid contour abnormalities such as notches, peaks, and flares, some studies have indicated that obtaining a symmetric MRD1 may not be sufficient for an acceptable result [3]. Eyelid contour abnormalities secondary to ptosis surgery may cause various complaints, including cosmetic problems, visual field defects, and ocular surface disorders [3]; researchers have thus directed their investigations to eyelid contour analysis [4–8]. However, surgical outcomes of ptosis related to eyelid contour have conventionally been evaluated based solely on subjective grading systems, such as excellent-good-acceptable scales [3, 7, 9]. Recently, Garcia et al. evaluated lower lid contour in patients with Graves orbitopathy [10]. Garcia et al. reported that they conducted this study to observe the effect of procedures on lower lid contour in the patients with Graves lower lid retraction. They evaluated the lower lid using NIH Image J (https://imagej.nih.gov/ij) and Matlab software. In a subsequent study, Golbert et al. described the degree of symmetry of upper lid contour in healthy subjects using the Bezier curve, named after engineer Pierre Bezier [11]. To build on the findings of this study performed on healthy subjects [11], we analyzed the symmetry of upper lids preoperatively and postoperatively in patients with unilateral ptosis. The primary purpose of this study was to assess outcomes of unilateral ptosis correction based on parameters including degree of symmetry, MRD1, peak height of the upper lid (PHUL), temporal and nasal ocular surface area, and temporal/nasal (T/N) area ratio with an objective, quantitative, and repeatable method.

## Methods

The present study was carried out in accordance with the Helsinki Declaration principles. The ethical committee of Izmir Tepecik Training and Research Hospital approved the study, and informed consent of all patients was obtained for taking clinical photographs and publishing data. This study was designed as a retrospective, non-randomized case-control study. Medical records of all patients with unilateral ptosis between October 2015 and December 2020 were reviewed. Patients with unilateral ptosis who underwent surgical correction and demonstrated levator function (LF) of 5 mm or higher were included in the study. Only the patients who underwent surgery under local anesthesia were included in the study. Two groups were defined: the ptotic eyes comprised the case group, and the contralateral eyes made up the control group. Patients with bilateral ptosis, additional concomitant eyelid malpositions, history of previous eyelid surgery, orbital disease, Graves ophthalmopathy, history of radiation, neuromuscular disease, facial palsy, and less than 6 months of follow-up were excluded. In addition, patients who underwent botulinum toxin type A injection within 5 months prior to ptosis surgery were excluded.

Demographic data, preoperative and postoperative clinical photographs, and MRD1, LF, Bell phenomena, and ocular motility were reviewed on patient files.

Ptosis severity was categorized into three groups: mild (1–2 mm), moderate (2–4 mm), and severe (greater than 4 mm). LF was classified as excellent, good, fair, poor, and none, with excellent corresponding to greater than 10 mm, good to 7–10 mm, fair to 5–7 mm, and poor to 1–4 mm of LF.

### Image analysis

Image processing was performed with the PubMed-sourced NIH Image J software (available at https://imagej.nih.gov/ij/), which has been used in multiple previous ophthalmologic studies [10–13]. Preoperative and postoperative MRD1, PHUL, distance between PHUL and MRD1, nasal and temporal ocular surface areas covered by the upper lid, and the horizontal line connecting the lateral and medial canthi were measured.

**Fig. 1:**
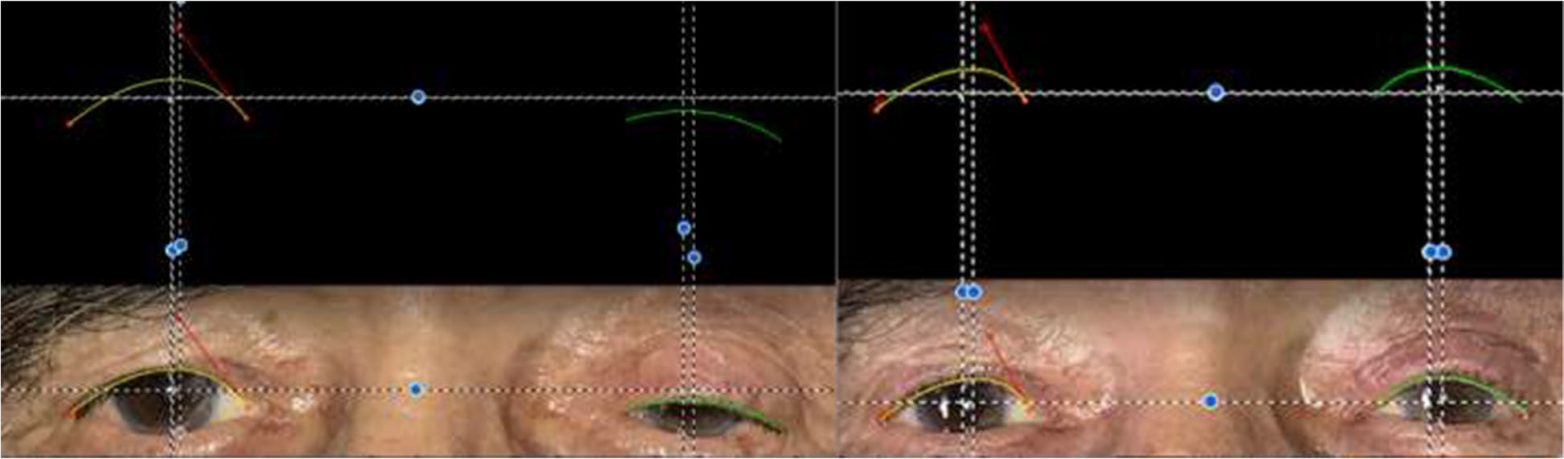
An example of a patient whose preoperative and postoperative clinical photographs was employed in Image J software and bilateral eyelid contours were extracted and coordinates of eyelid parameters were obtained. Using vertical and horizontal lines passing through corneal light reflex, pupil center, MRD1, peak of the upper lid, temporal and nasal ocular surface areas are easily identifiable

During image processing, head rotation was first corrected if needed by aligning the bilateral canthi using the transform and rotate component of the software. As described in the study by Golbert et al. [11], utilizing the Bezier curve tool, two control points, one located on the lateral canthus and the other on the upper punctum, were assigned. Dragging these control points, a curve completely fitting to the upper eyelid contour was drawn. Two vertical lines passing through the MRD1 of each eye were drawn. If the PHUL was located medial or lateral to the mid-pupil, a vertical line was added corresponding to the PHUL. A transverse line passing through the bilateral MRD1 was added. Thereby, each eye was separated by vertical and transverse lines to evaluate and compare the mathematical relations of the eyelid parameters. (Fig. 1) The distance between the peak height and central height of the upper lid was determined according to the coordinate system: if the peak and central height lines overlapped, the line was assigned a value of 0; if the peak was located medial to the central height, the distance was given a negative value; and if the peak was located lateral to the central height, the distance was given a positive value.

**Fig. 2:**
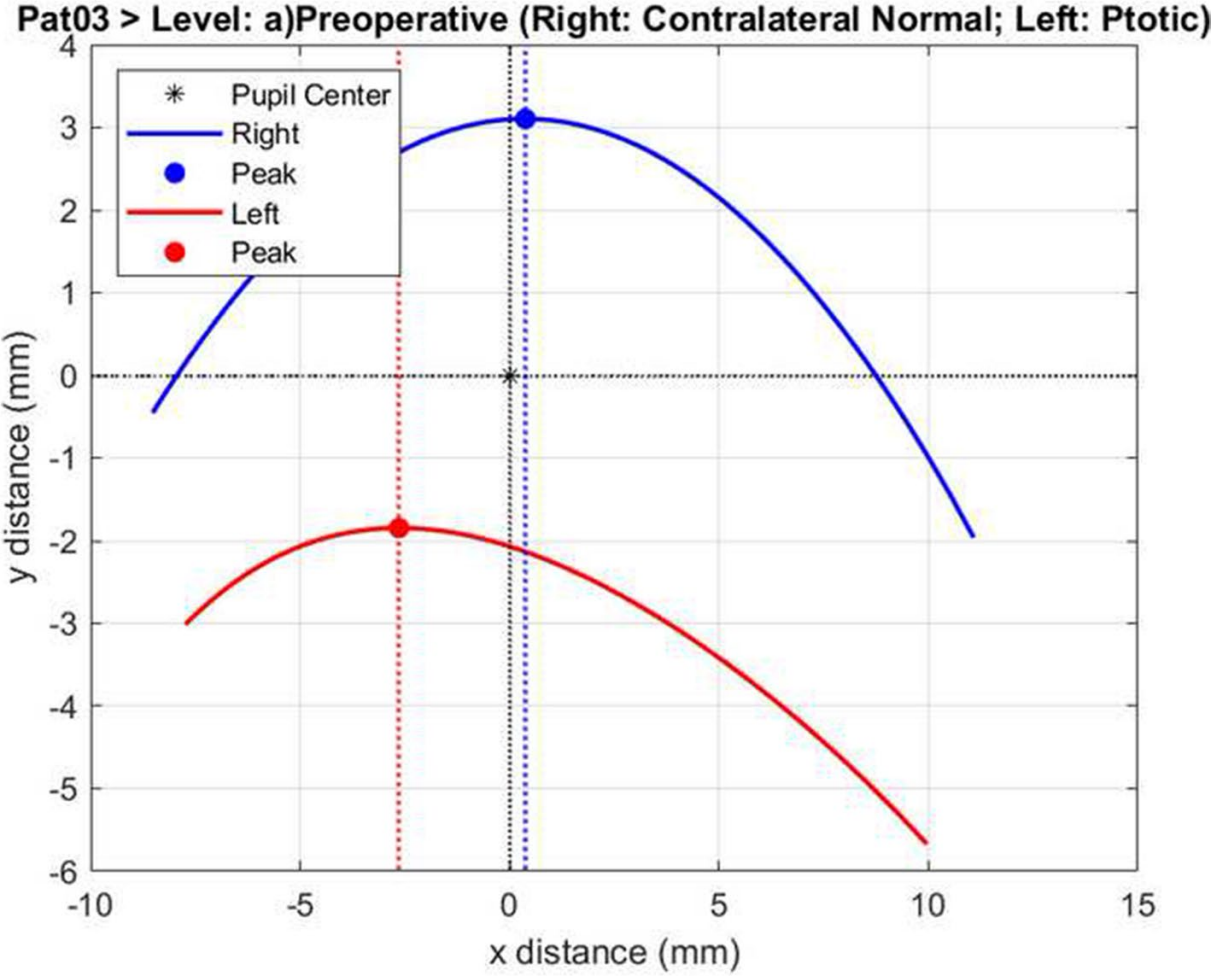
This figure is representing preoperative overlapping of ptotic and contralateral normal eye. (Data of the same patient in Fig. 1 was used for Figs. 2 and 3.) Similarity is 1.15% in this preoperative evaluation

**Fig. 3:**
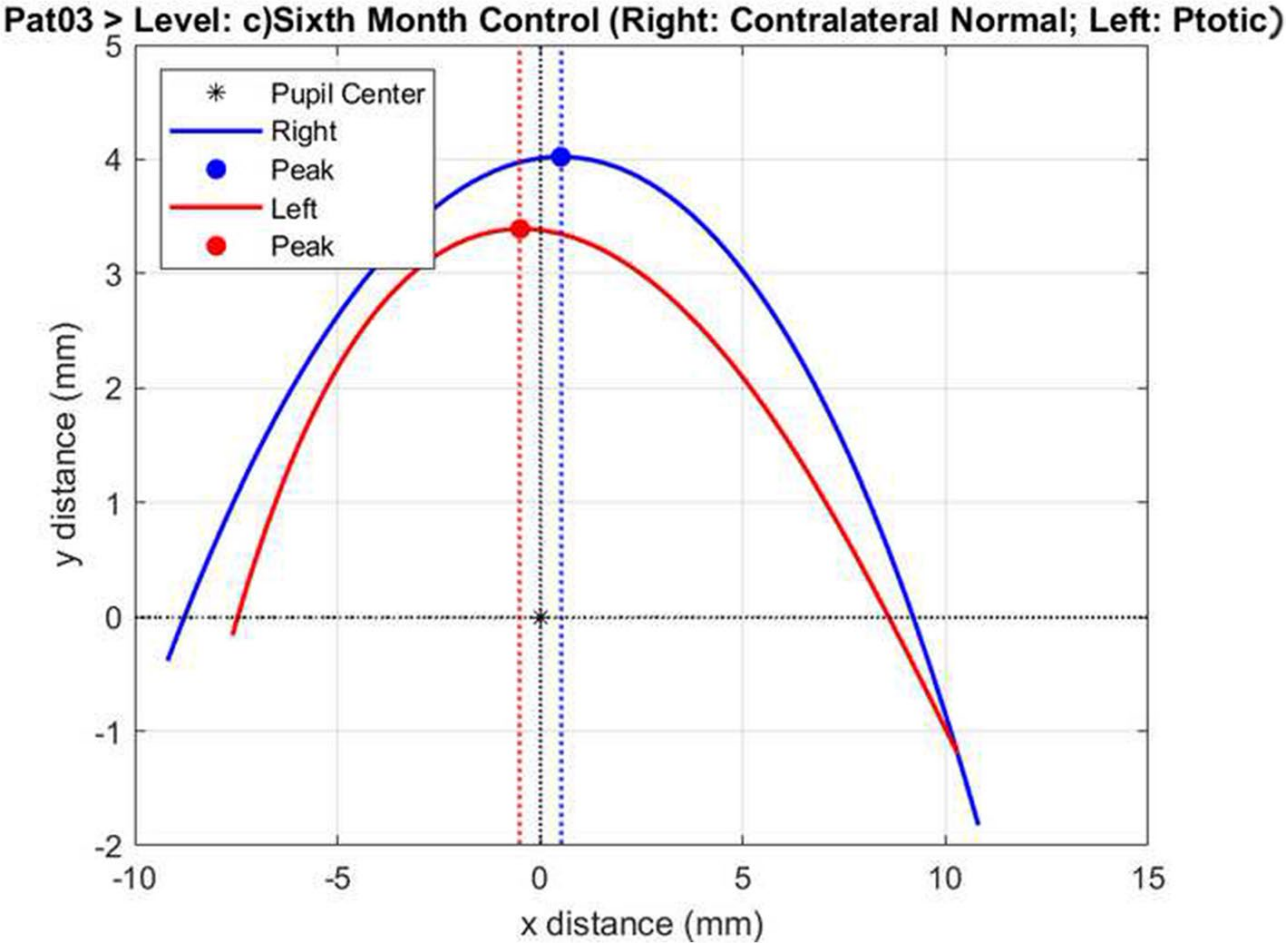
This figure is representing the same patient’s postoperative overlapping of ptotic and contralateral normal eye. Similarity is 78.5%

**Fig. 4:**
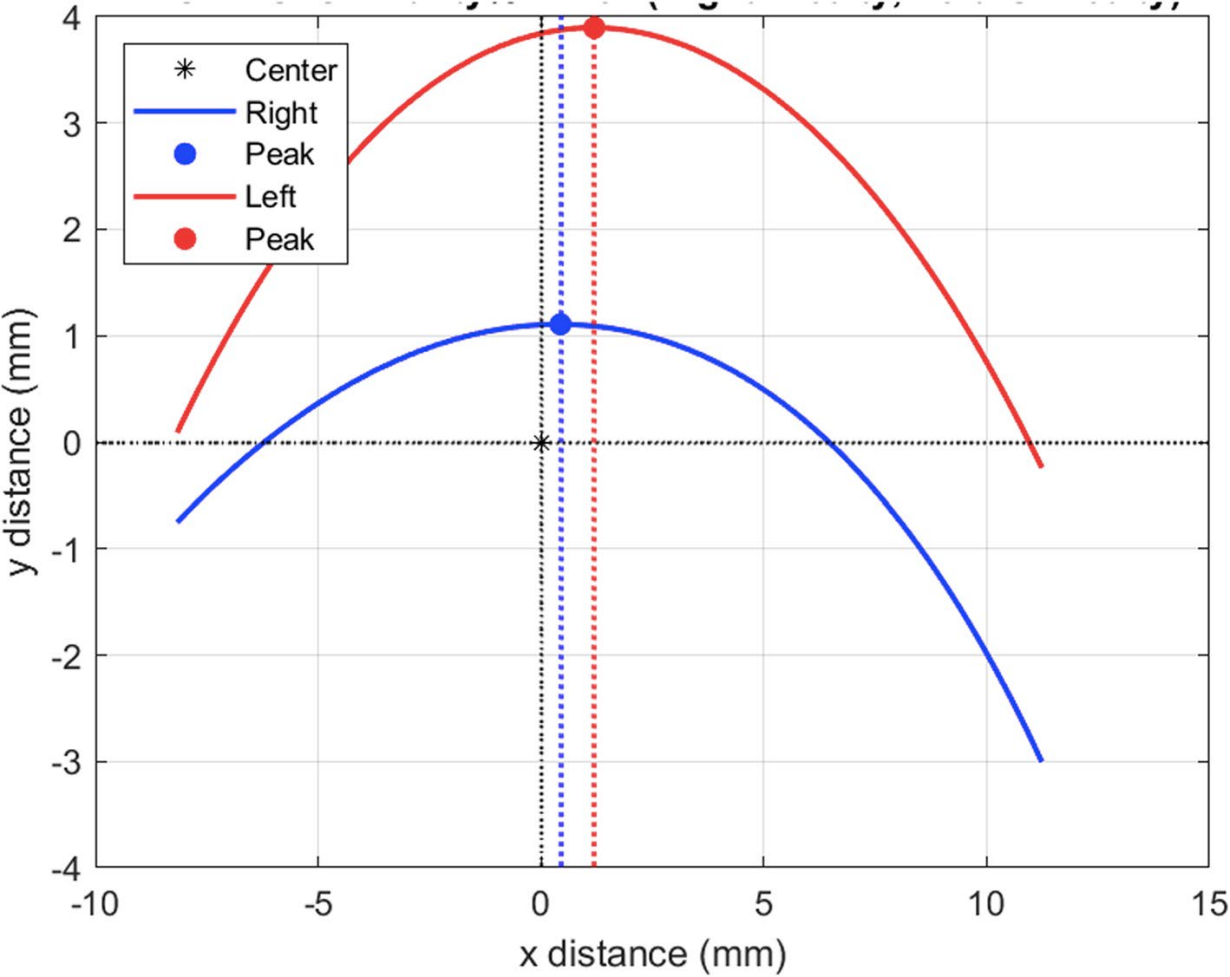
Representing 25% degree of symmetry, this figure was added for the purpose of demonstration

Using image processing with the threshold function, eyelid contour was extracted. Coordinates of the curve representing the extracted eyelid contour and corneal light reflex were saved and transferred to the Matlab software (Matlab, Mathworks, Inc., Natick, MA). The coordinates were used to determine the curve of the upper lids for each ptotic and contralateral eye. The curve lines were resampled to contain 1000 points and smoothed with a Savitzky–Golay filter. The curve of the operated eye was flipped from lateral to medial to overlay the ptotic and contralateral eye curves. The corneal light reflex was used as the reference coordinate for overlaying. Contour symmetries were defined according to the degree of overlap of the two curves. Using the Matlab software, the degree of symmetry was evaluated for both total eyelid contour and medial and lateral portions of the upper lid (Figs. 2, 3 and 4).

### Surgical technique

The ptosis correction procedure was performed on 34 patients under local anesthesia, with a 2 mL local anesthetic containing 40 mg lidocaine and 0.025 mg epinephrine injected into the lid crease 10 min before surgery. A skin incision was made through the lid crease, the orbicularis oculi was dissected, and the tarsal plate was found. The orbital septum was opened and the levator superioris muscle was identified. A horizontal lamellar bite through the central third of the upper tarsal plate was performed. A temporary knot was placed to bind the levator superioris muscle to the tarsal plate, and the patient was moved to a sitting position to inspect the height and contour of the upper eyelid. After adjusting the height and contour, a 6/0 absorbable polyglactin suture was used to attach the levator superioris muscle to the tarsal plate. Three sutures, comprising one central cardinal, one lateral, and one medial, were placed. Levator resection was performed in the patients with congenital ptosis, and a combination of levator resection and advancement was performed for the other patients. Lateral and medial horns of the levator aponeurosis were preserved in both resection and advancement surgeries. A strip of orbicularis oculi and skin were removed if required. The skin was then closed with a 6/0 Prolene suture.

### Statistical analysis

Data were analyzed using Statistical Package for Social Sciences version 16 (SPSS Inc., Chicago, III., USA). Shapiro–Wilk and Kolmogorov–Smirnov tests were used to assess the normality of the data. Data demonstrating normal distribution were compared using the independent t-test and paired t-test, while data not demonstrating normal distribution were compared with Mann–Whitney U and Wilcoxon tests. A *p* value of < 0.05 was considered statistically significant.

## Results

Of 34 patients, 19 were female and 15 were male. None of the patients had strabismus, and the eye movements of all patients were recorded as normal in every direction. Mean patient age was 58.8 ± 12.7 years (range 15–75 years). Mean follow-up time was 19.5 ± 7.3 months (range 8–40 months). Four patients were diagnosed with congenital ptosis and 30 patients were diagnosed with aponeurotic ptosis.

Mean preoperative MRD1 was 0 ± 1.14 mm (range − 2 to 3 mm) for ptotic eyes and 3.3 ± 0.4 mm (range 3–4.2 mm) for contralateral eyes. Moderate ptosis was found in 22 patients and severe ptosis was found in 12 patients. LF was excellent in 20 patients and good in 14 patients. Mean preoperative PHUL was 0.45 ± 1.11 mm (range − 1.5 to 3.1 mm) for ptotic eyes and 3.4 ± 0.36 mm (range 3–4.2 mm) for contralateral eyes. Mean preoperative distance between MRD1 and PHUL for the ptotic group was − 0.03 ± 1.57 mm (range − 4 to 2 mm), while it was 1.2 ± 1.5 mm (range − 1 to 4 mm) for the control group. Mean MRD1, PHUL, and distance between MRD1 and PHUL for preoperative, postoperative, and 6-month follow-up time points are presented in Table 1.

**Table 1:** Mean MRD1, Peak height of upperlid, distance between MRD1 and peak height of upper lid values for preop and postop first and sixth month

	Preop	Postop firsth month	Postop 6th month
MRD1 for ptotic group	0 ± 1.14 mm	3.26 ± 0.55 mm	3.33 ± 0.53 mm
MRD1 for control group	3.3 ± 0.34 mm	3.25 ± 0.30 mm	3.27 ± 0.28 mm
PHUL for ptotic group	0.49 ± 1.11 mm	3.43 ± 0.91 mm	3.41 ± 0.7 mm
PHUL for control group	3.39 ± 0.36 mm	3.33 ± 0.31 mm	3.37 ± 0.33 mm
Distance between MRD1-PHUL in ptotic group	−0.03 ± 1.57	1.96 ± 1.41 mm	1.87 ± 1.41 mm
Distance between MRD1-PHUL in control group	1.25 ± 1.55 mm	1.22 ± 1.47 mm	1.3 ± 1.49 mm

Preoperatively, mean nasal area was 9.1 ± 9.9 mm^2^ and mean temporal area was 10.5 ± 9 mm^2^ for the ptosis group, while mean nasal area was 31.2 ± 4.3 mm^2^ and mean temporal area was 37.1 ± 4.5 mm^2^ for the control group. T/N area ratio for contralateral eyes was 1.19 preoperatively and postoperatively; it was 1.11 preoperatively and 1.15 postoperatively for operated ptotic eyes. Mean preoperative and postoperative temporal and nasal areas and T/N ratios for contralateral and ptotic eyes are displayed in Table 2. There was no significant difference between preoperative and postoperative T/N ratios (*p* = 0.78).

**Table 2:** Mean nasal,temporal ocular surfaces areas and T/N ratios values for preop and post-op first and sixth month

	Pre-op	Post-op first month	Post-op sixth month
Nasal area for ptosis group	9.1 ± 9.9 mm^2^	33.04 ± 6.8 mm^2^	31.2 ± 6.7 mm^2^
Nasal area for control group	31.2 ± 4.3 mm^2^	30.9 ± 3.2 mm^2^	31.3 ± 4.1 mm^2^
Temporal area for ptosis group	10.5 ± 9 mm^2^	38.38 ± 7.2 mm^2^	36.3 ± 7.4 mm^2^
Temporal area for control group	37.1 ± 4.5 mm^2^	37.4 ± 3.8 mm^2^	37.2 ± 4.2 mm^2^
T/N area ratio for control group	1.19	1.19	1.19
T/N area ratio for ptosis group	1.11	1.15	1.16

Mean preoperative degree of symmetry for overall eyelid contour was 36.6% ± 27.5% (range 1–92%). Mean preoperative degree of symmetry for nasal eyelid contour was 41% ± 30.1% (range 0–95%), and for temporal eyelid contour, the preoperative degree of symmetry was 33% ± 27.5% (range 2–90%). Mean preoperative and postoperative degrees of symmetry are presented in Table 3. Preoperatively, the degree of symmetry was 20% in patients with severe ptosis and 45% in patients with moderate ptosis. Postoperatively, the mean degree of symmetry was 75% in patients with moderate ptosis, it was 68% in patients with severe ptosis. (See supplementary information to analyse the various degree of symmetry from 5 to 95%. Additional files 1, 2, 3, 4, 5, 6, 7, 8, 9, 10 and 11) There was a significant difference in degrees of symmetry between patients with severe and moderate ptosis before and after surgery (*p* = 0.012). There were no significant differences in T/N area ratio between patients with moderate and severe ptosis before and after surgery (*p* = 0.89) or between patients with excellent and good LF before and after surgery (*p* = 0.89). While there was no significant difference in the preoperative degree of symmetry between patients with excellent and good LF (*p* = 0.79), there was a significant difference after ptosis correction (*p* = 0.03).

**Table 3:** Degree of symmetry values of overall, temporal and nasal portion of eyelid for preop, post-op first and sixth month

*EYELID CONTOUR*	*DEGREE OF SYMETRY- %*		
	Pre-op	Post-op first-month	Post-op 6th-month
*OVERALL*	36	67	72
*NASAL*	41	69	70
*TEMPORAL*	33	68	71

There was a significant difference between preoperative and postoperative measurements of the distance between MRD1 and PHUL in the ptosis group (*p* < 0.001). There were also statistically significant differences in degree of symmetry between preoperative and initial postoperative and preoperative and 6-month follow-up results (*p* < 0.001 and *p* < 0.001, respectively). There was no statistical significance in the degree of symmetry between initial postoperative and 6-month follow-up results in the ptosis group (*p* = 0.86). There was no statistical significance in MRD1 between the postoperative ptosis group and the control group (*p* = 0.82) Regarding the impact of the age, and sex, on MRD1, PHUL, T/N ratio and degree of symmetry, we did not detect a correlation.

## Discussion

As MRD1 is insufficient to evaluate outcomes of ptosis correction, researchers have attempted to identify more comprehensive, objective, and quantitative assessment methods. Eyelid contour analyses have been performed with various methods, including mathematical polynomial functions, image processing, and mathematic software. Mocan et al. extracted eyelid contours using third-degree equations of polynomial functions [6]. Cruz et al., Akaishi et al., and Şendül et al. used T/N distances and area ratios [4, 5, 8], while Ahn et al. and Ribeiro et al. drew 12 oblique lines from the corneal reflex to the upper lid margin [7, 14] and Danesh et al. determined 10 reference points on the upper lid margin to compare the symmetry of the upper lid [3]. In a recent study, Golbert et al. reported the degree of symmetry between right and left upper lids in healthy subjects [11]. They used the Bezier curve function of the Image J software and produced 1000 reference points on the upper lid with approximately 0.03 mm spatial resolution. We employed the same protocol in this study to extract the upper lid contour and evaluate the degree of symmetry. Using Bezier curves, we compared the degrees of symmetry preoperative and postoperatively and calculated temporal and nasal areas. Golbert et al. reported a 96.1% degree of symmetry for the whole eyelid in healthy subjects and did not find any differences between the temporal and nasal portions of the lid [11]. In this study, the degrees of symmetry for the whole lid, temporal portion, and nasal portion preoperatively were 35.6, 32, and 42%, respectively, and postoperatively were 72, 72.3, and 71.2%, respectively. We could not achieve the expected postoperative MRD1 in two patients because of under-correction of the ptosis. Although the MRD1 showed a success rate of 94% in all patients, the overall degree of symmetry was only 72%. Differing success rates based on MRD1 and degree of symmetry supports that assessing MRD1 only may be insufficient to determine the success of outcomes. MRD1 and degree of symmetry were not different in the first and sixth months post-surgery in this study; first-month outcomes were consistent with sixth-month outcomes.

After ptosis correction, we detected a higher degree of symmetry in the patients with moderate ptosis than the patients with severe ptosis. The milder the ptosis, the greater the degree of symmetry achieved after ptosis correction. However, the severity of ptosis had no impact on the T/N ratio in this study. We also detected a higher degree of symmetry in the patients with excellent LF than the patients with good LF; while the mean degree of symmetry was 75% in the patients with excellent LF, it was 68% in the patients with good LF. We observed that higher preoperative LF was related to higher postoperative degrees of symmetry. However, LF had no impact on T/N ratio in this study.

Recent studies have reported a more temporal peak of the upper lid than previously reported [15], both in healthy subjects [16, 17] and patients with thyroid ophthalmopathy [18]. In addition, multiple studies have reported a slight temporalization of the peak after ptosis correction [7, 17, 19, 20]. In our study, while the mean peak of the contralateral eye was found to be 1.25 ± 1.55 mm temporal to the center of the pupil, the preoperative mean peak of the ptotic eye was located 0.03 ± 1.57 mm nasal and the postoperative mean peak of the ptotic eye was located 1.87 ± 1.41 mm temporal to the center of the pupil. These findings indicate that our intervention resulted in the temporalization of the peak. Considering the temporally located peak of the contralateral eye, we suggest that the postoperative temporalization of the peak of the ptotic eye contributed to the degree of symmetry. Even when ptosis led to a total drop of the eyelid, in this study, we quantitatively found an asymmetrically greater temporal drop of the eyelid. While the preoperative temporal degree of symmetry was 32%, the nasal degree of symmetry was 42%. The temporal drop of the lid was more significant in the patients with severe ptosis than those with mild and moderate ptosis. Considering the asymmetrical temporal drop and temporalization of the peak after surgery, the outcomes of this study are clinically consistent with the findings of cadaver studies, which reported that the lateral horn of the levator muscle is much stronger than the medial horn, and the lateral horn has an anatomic superiority to achieve the normal contour of the upper eyelid [21–23].

Previous studies have reported a T/N area ratio of 0.8–1.3, and T/N ratio is accepted as an indicator of eyelid contour symmetry [5, 8, 14]. In our study, T/N ratios were consistent with previous studies. The mean T/N ratio of the contralateral eye was 1.19, and in the ptotic eye, it was 1.11 preoperatively and 1.15 postoperatively. There was no significant difference between preoperative and postoperative T/N ratios for both ptotic and contralateral eyes. Considering these similar T/N ratios, it may be mistakenly concluded that postoperative contour symmetry was achieved or that preoperative symmetry was already present. However, the preoperative degree of symmetry was 35% and the postoperative degree of symmetry was 68%. T/N ratios were thus inconsistent with the degree of symmetry. These results suggest that the T/N ratio may not be a reliable determinant of eyelid contour symmetry. This discrepancy may have emerged as a result of disregarding the third dimension and calculating ocular surface area in only two dimensions.

This study has some limitations that warrant discussion. The primary limitation is the study’s retrospective nature, which limits the ability of the study’s outcomes to be applied to preoperative clinical decision-making. The study group was mostly comprised of patients with severe ptosis, which complicated the identification of the center of the pupil. Finally, all measurements were made on two-dimensional photographs rather than more accurate three-dimensional images.

## Conclusions

In conclusion, this study demonstrated a quantitative, objective, repeatable method to investigate the degree of symmetry after ptosis correction. In the case of unilateral ptosis, surgeons can determine contralateral eyelid contour parameters, including temporal–nasal portions, MRD1, and the peak of the upper lid. Thus, this method may help to improve surgical outcomes. In addition, using this method, surgeons can retrospectively evaluate the impact of their surgical interventions on the eyelid peak. Future prospective studies are needed to demonstrate the preoperative utility of this method. We evaluated the success of ptosis correction by degree of symmetry and MRD1 separately. Furthermore, this study concluded that the T/N ratio may not be a reliable parameter for evaluating eyelid symmetry. Therefore, future studies may consider an evaluation system that combines the degree of symmetry and MRD1 for a more accurate outcome assessment.

## Supplementary Information

**Additional file 1.** Demonstrating 5% degree of symmetry.

**Additional file 2.** Demonstrating 13% degree of symmetry.

**Additional file 3.** Demonstrating 20% degree of symmetry.

**Additional file 4.** Demonstrating 30% degree of symmetry.

**Additional file 5.** Demonstrating 45% degree of symmetry.

**Additional file 6.** Demonstrating 58% degree of symmetry.

**Additional file 7.** Demonstrating 68.5% degree of symmetry.

**Additional file 8.** Demonstrating 70% degree of symmetry.

**Additional file 9.** Demonstrating 83% degree of symmetry.

**Additional file 10.** Demonstrating 90.5% degree of symmetry.

**Additional file 11.** Demonstrating 95% degree of symmetry.

## Data Availability

The datasets used and/or analysed during the current study are available from the corresponding author on responsible request.

## Abbreviations

MRD1: Margin-Reflex Distance 1
LF: Levator Function
PHUL: Peak height of upper lid
T/N: Temporal and nasal ocular surface area ratio
