# Supplementary figures and images for: Comparing the symmetry of upper eyelid following unilateral ptosis correction
Source: BMC Ophthalmol. 2021 Dec 20;21:438. doi: 10.1186/s12886-021-02208-7 (PMC8686600; doi:10.1186/s12886-021-02208-7)

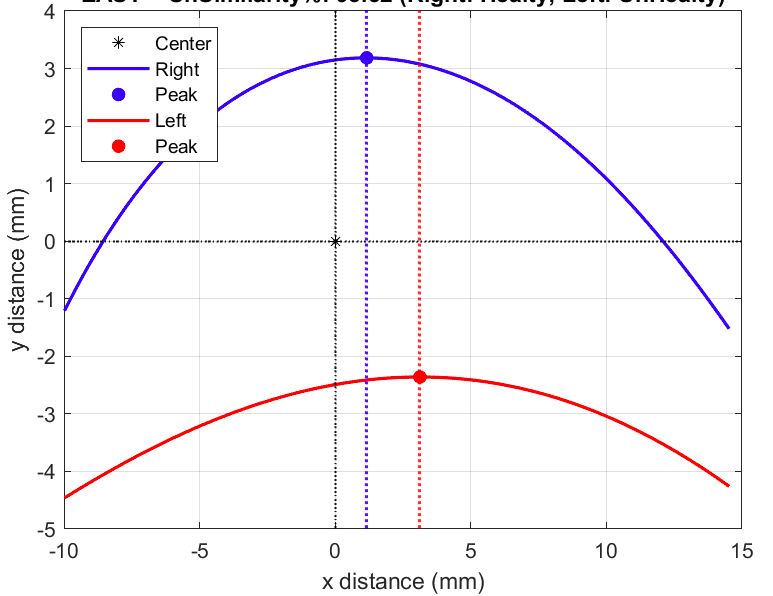

Supplement: Supplementary file 1 — Additional file 1. Demonstrating 5% degree of symmetry. [file 12886_2021_2208_MOESM1_ESM.png]

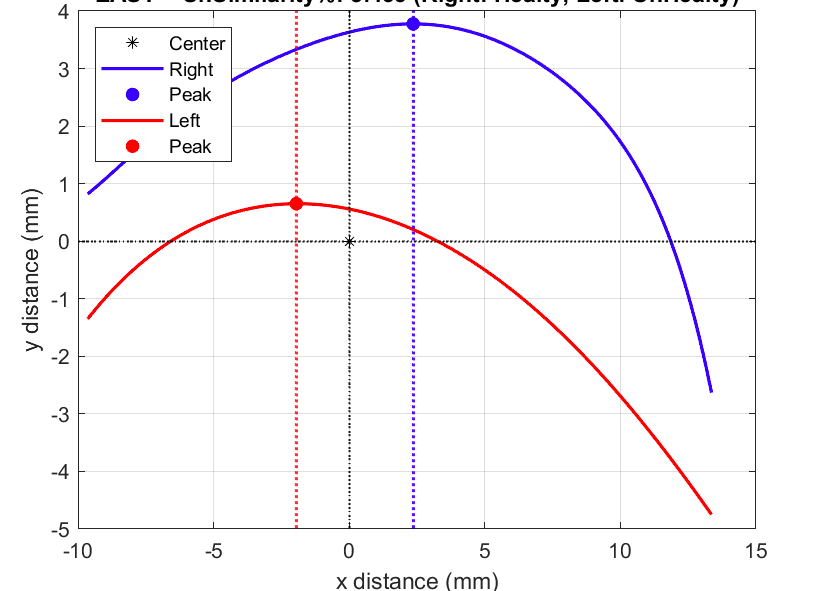

Supplement: Supplementary file 2 — Additional file 2. Demonstrating 13% degree of symmetry. [file 12886_2021_2208_MOESM2_ESM.png]

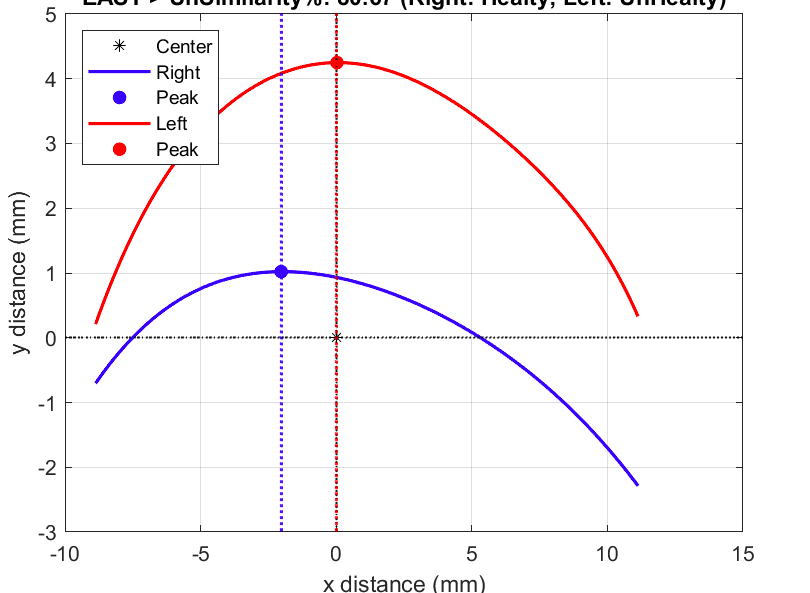

Supplement: Supplementary file 3 — Additional file 3. Demonstrating 20% degree of symmetry. [file 12886_2021_2208_MOESM3_ESM.png]

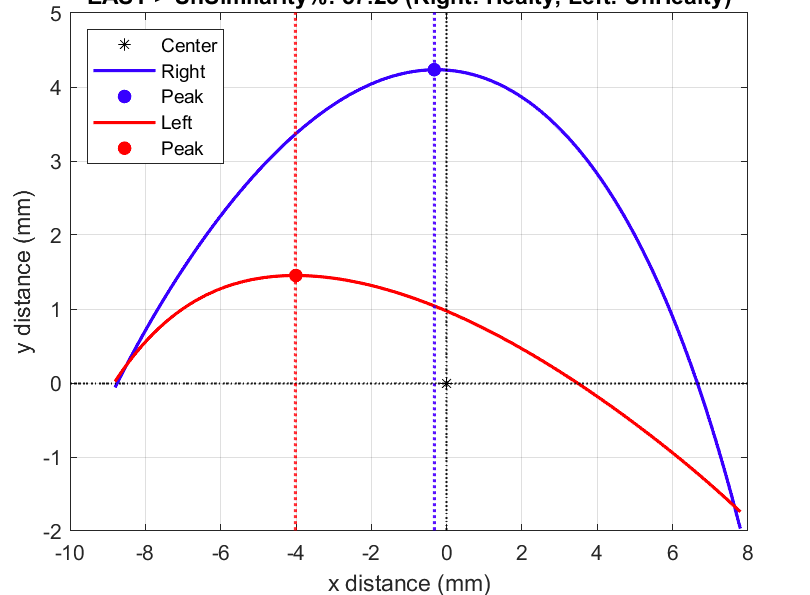

Supplement: Supplementary file 4 — Additional file 4. Demonstrating 30% degree of symmetry. [file 12886_2021_2208_MOESM4_ESM.png]

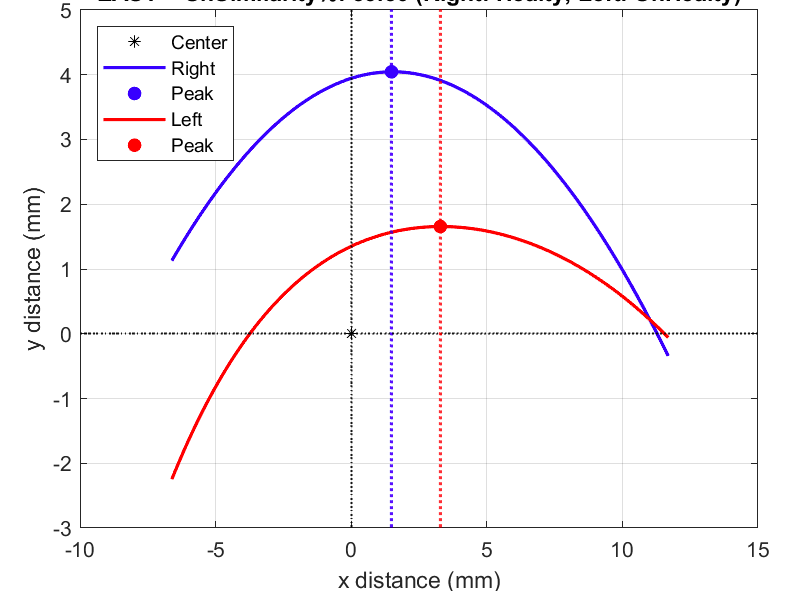

Supplement: Supplementary file 5 — Additional file 5. Demonstrating 45% degree of symmetry. [file 12886_2021_2208_MOESM5_ESM.png]

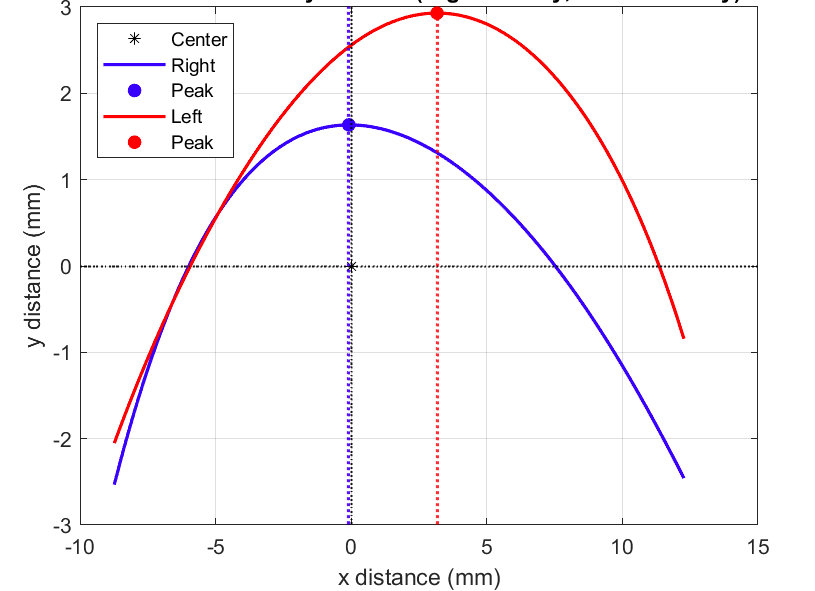

Supplement: Supplementary file 6 — Additional file 6. Demonstrating 58% degree of symmetry. [file 12886_2021_2208_MOESM6_ESM.png]

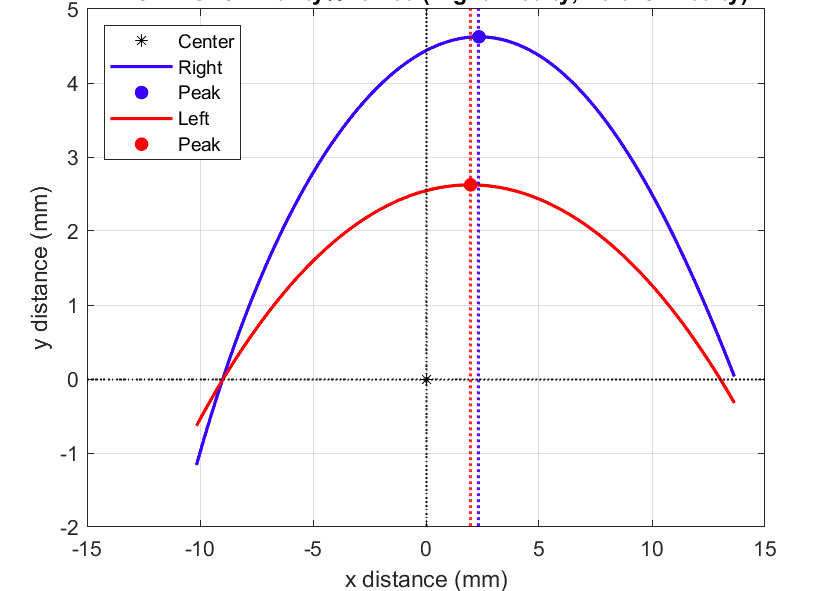

Supplement: Supplementary file 7 — Additional file 7. Demonstrating 68.5% degree of symmetry. [file 12886_2021_2208_MOESM7_ESM.png]

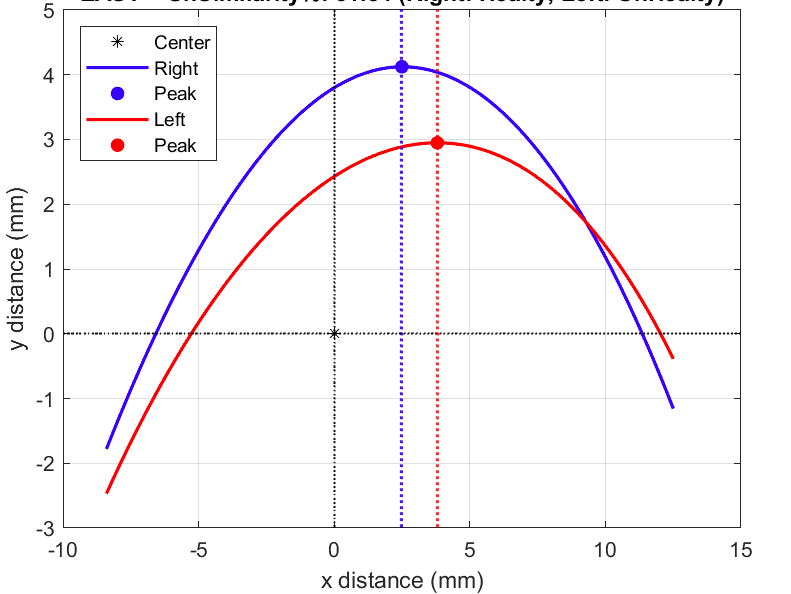

Supplement: Supplementary file 8 — Additional file 8. Demonstrating 70% degree of symmetry. [file 12886_2021_2208_MOESM8_ESM.png]

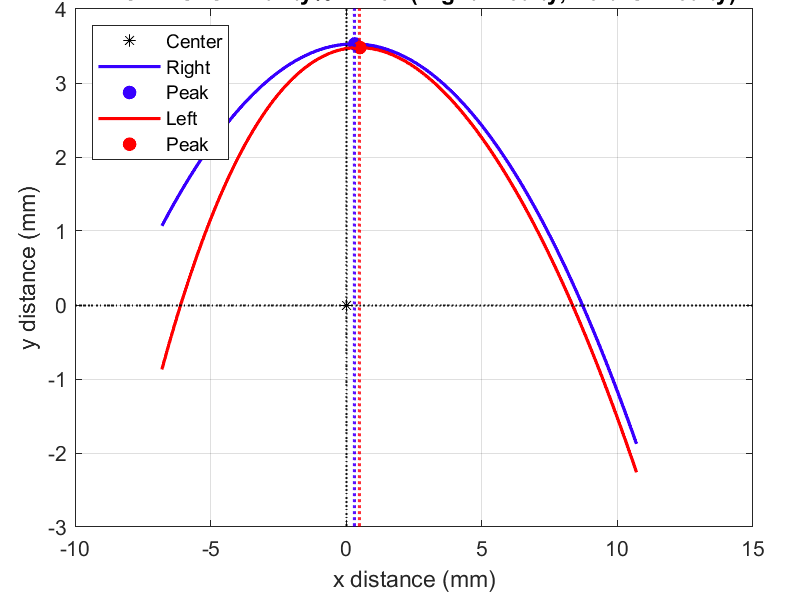

Supplement: Supplementary file 9 — Additional file 9. Demonstrating 83% degree of symmetry. [file 12886_2021_2208_MOESM9_ESM.png]

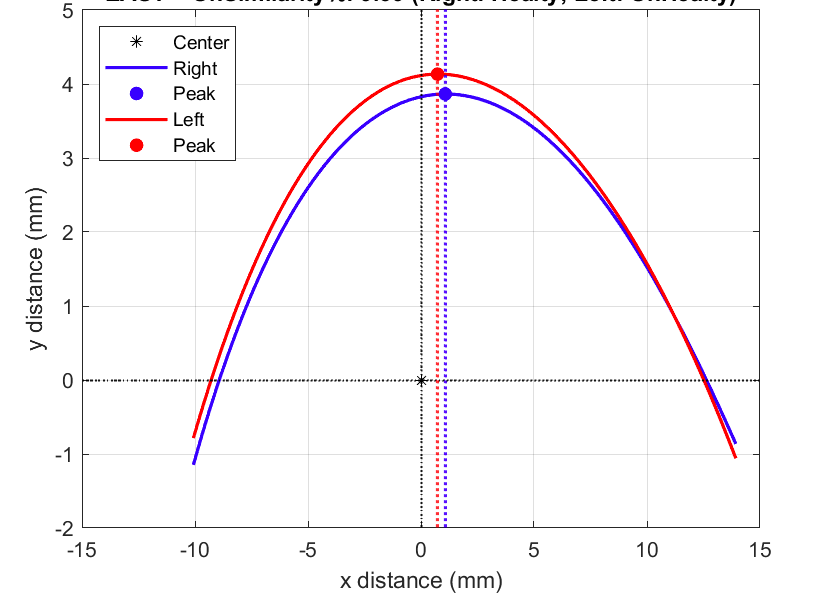

Supplement: Supplementary file 10 — Additional file 10. Demonstrating 90.5% degree of symmetry. [file 12886_2021_2208_MOESM10_ESM.png]

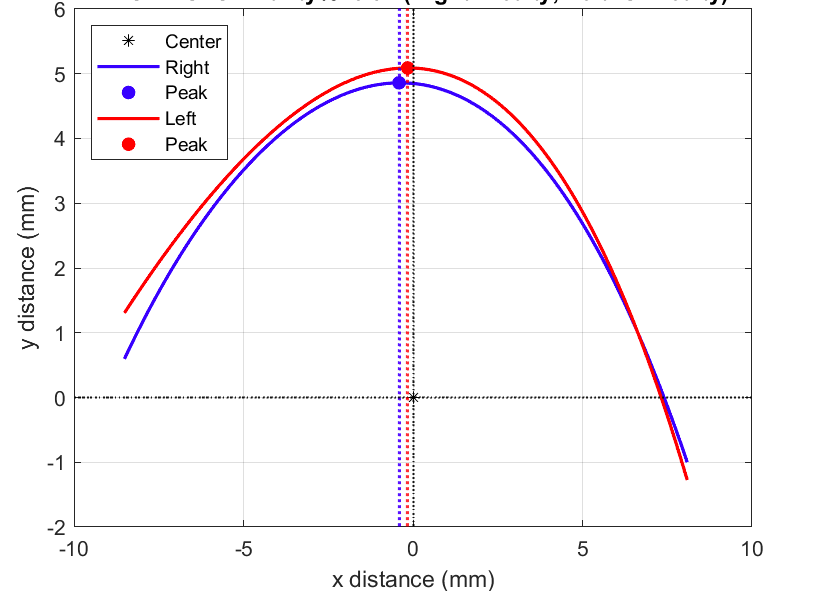

Supplement: Supplementary file 11 — Additional file 11. Demonstrating 95% degree of symmetry. [file 12886_2021_2208_MOESM11_ESM.png]
